# A New Member of the TBC1D15 Family from *Chiloscyllium plagiosum*: Rab GTPase-Activating Protein Based on Rab7 as a Substrate

**DOI:** 10.3390/md13052955

**Published:** 2015-05-13

**Authors:** Yuanyuan Li, Weidong Wang, Dandan Cheng, Tao Wang, Conger Lu, Jian Chen, Zuoming Nie, Wenping Zhang, Zhengbing Lv, Wutong Wu, Jianhong Shu

**Affiliations:** 1 College of Life Sciences, Zhejiang Sci-Tech University, Hangzhou 310018, China; E-Mails: liyy_08@163.com (Y.L.); wangweidong0106@163.com (W.W.); ddCzstu1990@126.com (D.C.); wangtao900121@163.com (T.W.); luconger0302@163.com (C.L.); chj1999@126.com (J.C.); wuxinzm@zstu.edu.cn (Z.N.); zwpcc@126.com (W.Z.); zhengbingl@zstu.edu.cn (Z.L.); wuwutong@gmail.com (W.W.); 2 Zhejiang Provincial Key Laboratory of Silkworm Bioreactor and Biomedicine, Hangzhou 310018, China

**Keywords:** APSL, TBC1D15 protein, Rab-GAP activity, type 2 diabetes

## Abstract

APSL (active peptide from shark liver) is a hepatic stimulator cytokine from the liver of *Chiloscyllium*. It can effectively protect islet cells and improve complications in mice with alloxan-induced diabetes. Here, we demonstrate that the APSL sequence is present in the *N*-terminus of novel TBC (Tre-2, Bub2 and Cdc16) domain family, member 15 (TBC1D15) from *Chiloscyllium* *plagiosum*. This shark TBC1D15 gene, which contains an ORF of 2088 bp, was identified from a cDNA library of regenerating shark liver. Bioinformatic analysis showed that the gene is highly homologous to TBC1D15 genes from other species. Moreover, the *N*-terminus of shark TBC1D15 contains a motif of unknown function (DUF3548), which encompasses the APSL fragment. Rab-GAP activity analysis showed that shark TBC1D15 is a new member of the TBC1D15 family. These results demonstrated that shark TBC1D15 possesses Rab-GAP activity using Rab7 as a substrate, which is a common property of the TBC1D15 family. The involvement of APSL at the *N*-terminus of TBC1D15 also demonstrates that this protein might be involved in insulin signaling and may be associated with the development of type 2 diabetes. The current findings pave the way for further functional and clinical studies of these proteins from marine sources.

## 1. Introduction

Hepatocyte stimulating factor (hepatic stimulator substance, HSS) was originally found in fetal liver or regenerating liver [1,2]. HSS can promote liver regeneration by stimulating DNA synthesis and mitosis in liver cells [3,4], resulting in the proliferation of liver cells and the repair of liver damage [5].

APSL (active peptide from shark liver) is an active factor similar to HSS that was first found in, and separated from, the regenerated liver of *Chiloscyllium* by Wu and colleagues in 2003 [5,6]. APSL has many functions, including immunoregulation, promoting hepatocyte growth, and repairing damaged islet β cells, which suggests its potential therapeutic effects in treating diabetes and liver damage [7]. Notably, APSL can play an auxiliary role in immunoregulation during the treatment of liver injury [8]. Huang *et al*. further showed that APSL has a hypoglycemic effect and protects islet cells in rats with streptozotocin (STZ)- or alloxan (ALX)-induced diabetes [9,10]. The mechanism underlying these features may be involved in the ability of APSL to enhance immunity, reduce the levels of cell inflammation factors and repair damaged islet β cells. APSL can also repair Ccl4-induced damage to hepatocytes by stabilizing the liver cell membrane, scavenging free radicals and inhibiting lipid peroxidation [6].

In a previous study, we expressed a CTB-APSL fusion protein using the *Bombyx mori* nucleopolyhedrovirus expression system in silkworm pupae [11]. A form of the CTB-APSL fusion protein suitable for oral dosage was then prepared. The pharmacodynamics associated with its oral administration was evaluated in an animal model of type 2 diabetes. A lyophilized powder made from silkworm pupae expressing the CTB-APSL fusion protein had a hyperglycemic effect against type 2 diabetes and decreased complications of diabetes [11]. Furthermore, the APSL sequence was found in the *N*-terminus of the shark TBC1D15 protein, a new member of the TBC domain family, member 15 (TBC1D15).

The TBC domain, recognized as a conserved protein sequence that consists of approximately 200 amino acid residues, exists in proteins in all eukaryotic cells [12,13]. The TBC domain functions as a GAP (GTPase-activating protein) for Rab proteins, which play important roles in vesicular trafficking pathways [14,15,16,17]. In general, there are two nucleotide-bound states in Rab cycles: GDP-bound (inactive) and GTP-bound (active). Interconversion between these two states is catalyzed by GEFs (guanine nucleotide exchange factors) and GAPs, which facilitate the exchange of GDP for GTP and accelerates the slow GTPase activity of Rab, respectively [14]. An increasing amount of evidence has shown that TBC proteins in mammals can recognize specific Rab proteins and participate in the biological activities of Rab proteins [18]. In addition, TBC1D15 exhibits a substrate preference for the Rab7 [14,19].

Cytoplasmic TBC1D15 is a highly conserved protein belonging to the TBC domain family and has similar functions to Rab-GAP. Only one TBC domain exists in the TBC1D15 proteins isolated from various species, and this domain is a conserved region containing six motifs (A–F); the diversity of TBC1D15 homologs is not obvious [12,13,14]. Importantly, the conserved arginine present in motif B is an essential amino acid for Rab-GAP activity [20,21]. In recent years, researchers have carried out a series of studies on TBC1D15. In 2010, Peralta *et al*. showed that as a Rab7-GAP, TBC1D15 could control lysosomal morphology and is very important for growth factor dependence [22]. In 2012, Feldman *et al*. found that TBC1D15 functions as an oncoprotein to regulate stem cell self-renewal [23]. Moreover, other researchers have shown that TBC1D15 in combination with Fis1 participates in the regulation of mitochondrial morphology [24].

In addition, some TBC1 family members have been reported as tools for regulating cell membrane trafficking events [13]. There is a very close relationship between TBC1 family proteins and insulin signal transduction [17,25,26]. During insulin stimulation, a large number of GLUT4 (glucose transporter type 4) proteins can be transported from intracellular vesicles to the cytoplasmic membrane [27,28,29,30]. Thus, the regulation of the GLUT4 vesicle transport mechanism has important implications for diabetes drug development. For example, studies have shown that TBC1D1 and TBC1D4 participate in intracellular GLUT4 vesicle transport and regulate blood glucose and that the site-specific phosphorylation of TBC1D4 is closely related to type 2 diabetes recovery [13,17,29,31].

In the present study, a miRNA library [32,33] and a cDNA library (data not shown) were established for the first time from regenerating shark liver tissue. Based on sequence alignment analysis between APSL sequences and the regenerating shark liver cDNA library, we identified a 4213 bp gene sequence containing 2088 bp of the ORF box as a novel TBC1D15 from *Chiloscyllium plagiosum*. This sequence is highly homologous to TBC1D15 genes from other species. *In vitro* GAP assays and interactions with Rab7a confirmed it as a new type of TBC1D15 from *C. plagiosum* with Rab-GAP activity for Rab7. Because the APSL fragment lies in the *N*-terminus of the shark TBC1D15 protein, we speculate that shark TBC1D15, containing the APSL fragment, may be closely associated with type 2 diabetes. This gene offers new possibilities for the development of potential anti-diabetic drugs from marine resources [34].

## 2. Results

### 2.1. Blast Analysis of the Shark TBC1D15 Genome Sequence

**Figure 1:**
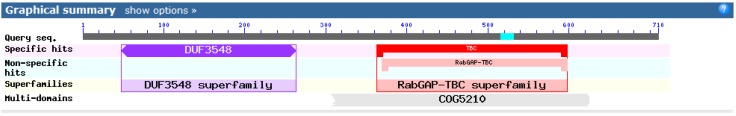
Functional domain analysis of shark TBC1D15. Shark TBC1D15 contains two domains, one from the DUF3548 superfamily and the other from the Rab-GAP-TBC superfamily. The DUF3548 domain is located in the APSL segment.

Sequence alignment analysis between the APSL sequences and the shark regenerating liver cDNA library identified a new TBC1D15 gene (4213 bp) containing an ORF of 2088 bp; the APSL sequence is located in the *N*-terminus of the novel shark TBC1D15 protein. Using the NCBI Blast software (http://blast.ncbi.nlm.nih.gov/Blast.cgi), we found several functional sites in the shark TBC1D15 protein sequence. Amino acids 48–263 form a DUF3548 functional domain containing the APSL fragment, and amino acids 366–576 form a TBC functional domain. The TBC domain has Rab-GAP activity (Figure 1), but the function of the DUF3548 domain remains unknown.

### 2.2. Homology Analysis of the Shark TBC1D15 Amino Acid Sequence

A Blastp comparative analysis against the NCBI databases showed that the amino acid sequence of the novel shark TBC1D15 gene is highly homologous to TBC1D15 from other species. The homology percentages between the shark TBC1D15 protein and TBC1D15 proteins from humans, chimpanzees, rats and mice are 67%, 66%, 65%, and 64%, respectively. Moreover, the homology between the TBC functional domain of the shark TBC1D15 and that of *Homo sapiens* is as high as 91%.

**Figure 2:**
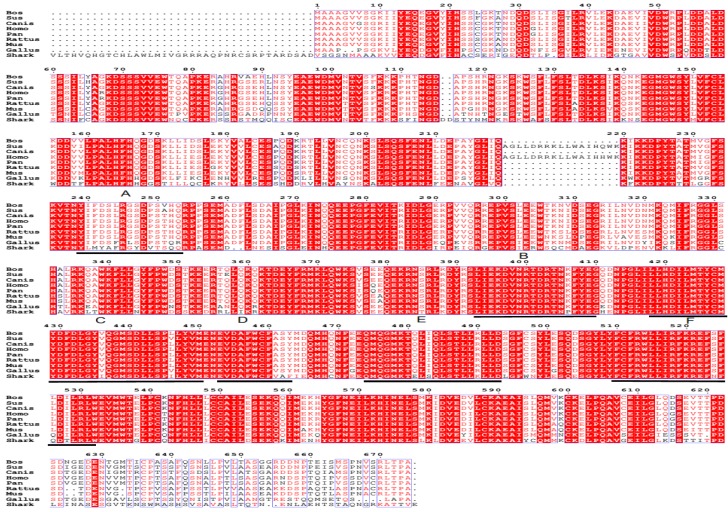
Alignment of the deduced amino acid sequence of the shark TBC1D15 with other homologous proteins. The sequential order of sequence alignment for the TBC1D15 homologs from nine species are as follows: *Bos*, *Sus*, *Canis*, *Homo*, *Pan*, *Rattus*, *Mus*, *Gallus*, and *Shark*. Six motifs (A–F) are underlined.

Based on the sequence homology, six typical sequence motifs (A–F) are present in shark TBC1D15; they are found in all members of the TBC1D15 family [12,14] and are highly conserved. Some residues of TBC1D15 are strictly conserved, such as Arg245 in motif A and Arg400 in motif B [14]. Consequently, we concluded that the novel gene we found in regenerating shark liver belongs to the TBC1D15 family. The results from the comparisons of homologous sequences and the six motif sites are shown in Figure 2.

### 2.3. Shark TBC1D15 Interacts with Rab7 in Vitro

**Figure 3:**
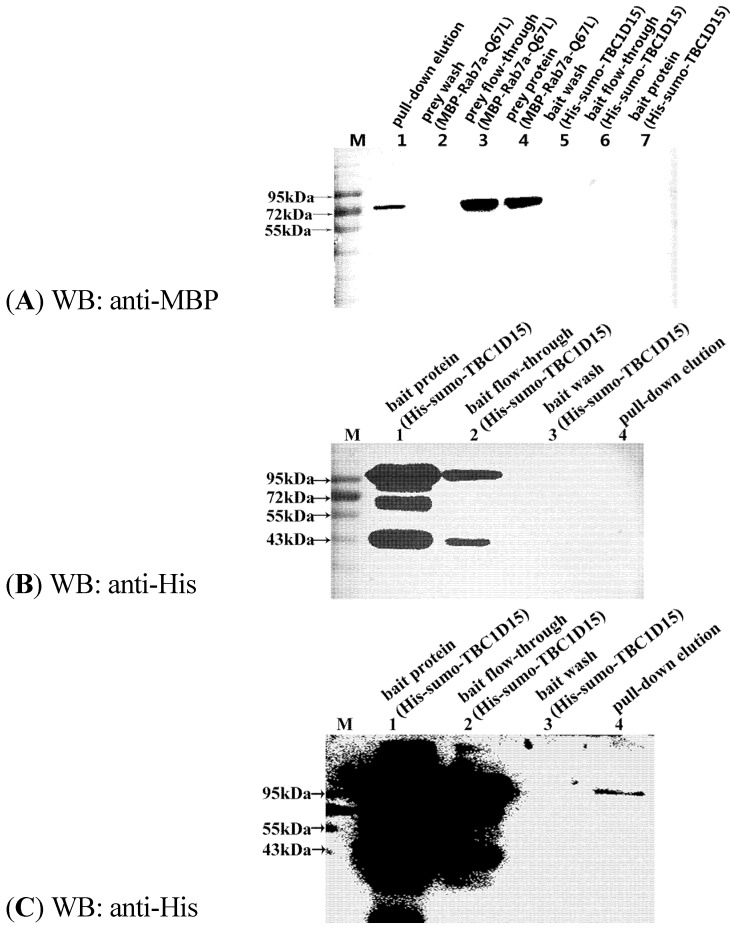

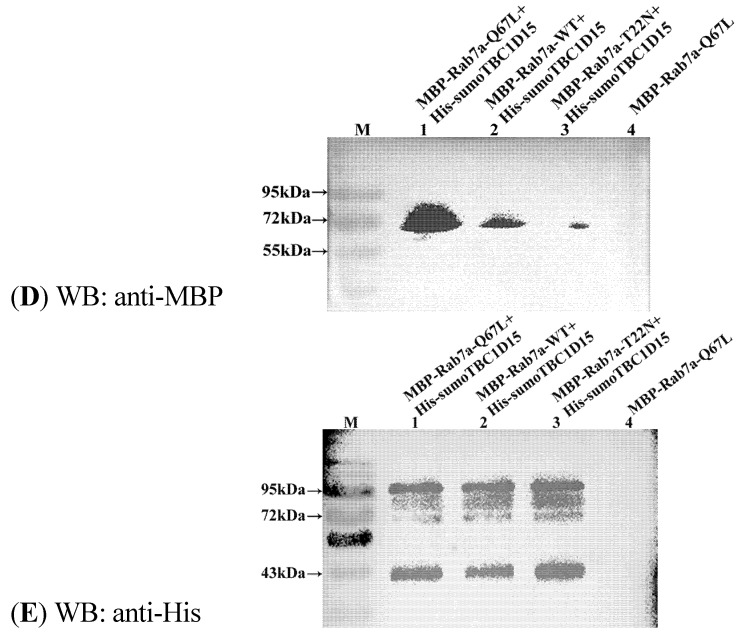
Interaction between shark TBC1D15 and Rab7a. Western blotting analysis of the pull-down elution using anti-MBP and anti-His antibody detection. (**B**,**C**) The same sample at different exposures. (**A**) Anti-MBP Western blot of the His-sumo-TBC1D15 and MBP-Rab7a-Q67L pull-down; (**B**,**C**): Anti-His Western blot of His-sumo-TBC1D15 and MBP-Rab7a-Q67L pull-down; (**D**) Anti-MBP Western blot of His-sumo-TBC1D15 and MBP-Rab7a-Q67L/WT/T22N pull-down; (**E**) Anti-His Western blot of His-sumo-TBC1D15 and MBP-Rab7a-Q67L/WT/T22N pull-down.

To demonstrate that shark TBC1D15 is a binding partner of the Rab7a protein, recombinant Rab7 protein was expressed and purified. Based on previous reports, Rab7a mutants representing the active, GTP-bound state (Rab7a-Q67L) and the inactive, GDP-bound state (Rab7a-T22N) were constructed using overlapping PCR and then expressed. Various forms of the recombinant Rab7a protein were purified to test the Rab-GAP activity of the shark TBC1D15 protein with pull-down experiments *in vitro*. To confirm whether this shark TBC1D15 is a new member of TBC1D15 with Rab-GAP activity and showing interaction with Rab7, we first tested its interaction with Rab7a-Q67L. Western blotting (WB) using anti-MBP and anti-His showed that this protein could bind to Rab7a-Q67L (Figure 3A–C). Then, an *in vitro* pull-down experiment was performed with this new TBC1D15 and Rab7a-WT/Q67L/T22N fusion proteins. During the pull-down experiment, equal amounts of His-sumo-TBC1D15 (150 µg) were first added to the #1, #2 and #3 nickel columns, followed by equal amounts (30 µg) of MBP-Rab7a-WT, Q67L and T22N, respectively. The #4 nickel column was used as a control, and the same amount of MBP-Rab7-Q67L was added without His-TBC1D15. The intensity of WB bands that appeared in the membrane was, from highest to lowest, Q67L→WT→T22N. No bands were detected in the control group (Figure 3D,E). In addition, the WB results suggested that the expression of His-sumo-TBC1D15 (95 kD) was unstable and that the protein could be easily fractured into peptides with molecular sizes of approximately 70 kD and 40 kD (Figure 3B).

### 2.4. In Vitro Analysis of the GAP Activity of Shark TBC1D15

Rab cycles between the GTP-bound and GDP-bound states. These cycles require two specific Rab effectors: GEFs and GAPs. Rab has a weak intrinsic ability to hydrolyze GTP, but a GAP can promote the rate of GTP hydrolysis by interacting with Rab. Previous research has shown that TBC1D15 is a Rab7-GAP [14]. We next sought to determine whether this novel shark TBC1D15 has Rab-GAP activity, a typical characteristic of TBC1D15 proteins.

**Figure 4:**
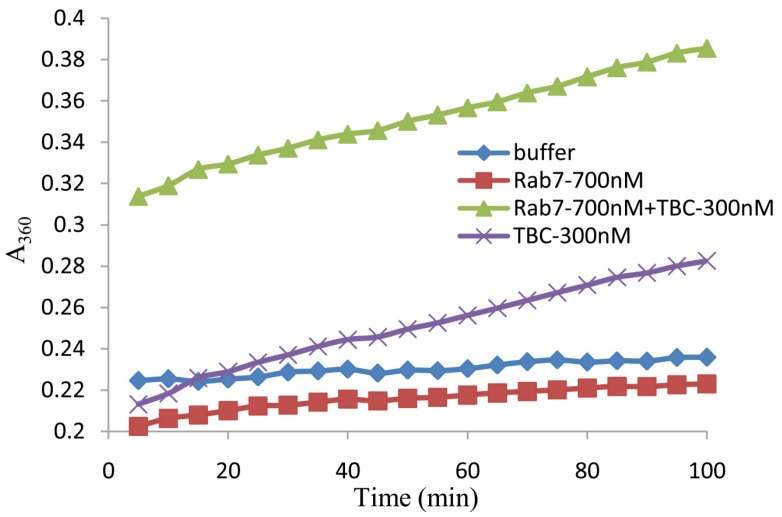
The release of inorganic phosphate (Pi) by GTP hydrolysis through an interaction between TBC1D15 and Rab7a-WT. The TBC1D15 protein shows Rab-GAP activity for Rab. GTP was used as the substrate for Rab. The blank control (buffer) was the only sample without any protein. The release of Pi was detected using a spectrophotometer at 360 nm (A_360_) every 5 min.

To quantify the GAP activity of the shark TBC1D15 *in vitro*, the recombinant proteins His-TBC1D15 and MBP-Rab7a-WT were expressed and purified. According to the instructions of the EnzChek^®^ Phosphate Assay Kit (Invitrogen E6646, Eugene, OR, USA), the absorbance at 360 nm (A_360_), which reflects the levels of inorganic phosphate (Pi), was detected using a spectrophotometer. The curve showed changes in the reaction between TBC1D15 and Rab7a-WT. When Rab or TBC1D15 was added, the Pi content was the same as the blank control (only buffer), which did not increase and instead remained constant. The Pi content of the experimental group (TBC1D15 + Rab7a) increased significantly (A_360_ increased from 0.3 to 0.4). These data indicate that the protein is a new TBC1D15 from shark and has Rab-GAP activity for Rab7 (Figure 4). 

## 3. Discussion

A cDNA library for regenerating shark liver has been established (data not shown). In the present study, a new TBC1D15 gene with a total length of 4213 bp containing an ORF of 2088 bp, which had originally been found in cartilaginous fish, was identified in the cDNA library using bioinformatics analysis. Surprisingly, the sequence of the APSL fragment matched the *N*-terminus of the shark TBC1D15 protein.

Blast analysis using the NCBI database showed that amino acids 48–263 in the *N*-terminus of the shark TBC1D15 protein serve as a DUF3548 functional domain, whereas amino acids 366–576 comprise a TBC functional domain. A domain with unknown function, DUF3548, contains the APSL peptide. The bioinformatics analysis indicated that the TBC1D15 gene from *Chiloscyllium* is highly homologous to that of other species, such as humans, chimpanzees, and mice. The TBC domain of TBC1D15 from *Chiloscyllium* exhibits the highest degree of homology (91%) to the TBC domain of *Homo sapiens*. However, it has yet to be proven that the protein is a novel TBC1D15 from *Chiloscyllium*.

Compared with other Rabs, human TBC1D15 has Rab-GAP activity and displays a substrate bias for Rab7a [14,35]. If the protein is a new shark TBC1D15 and its TBC domain has the highest homology with human TBC1D15, this protein will interact with human Rab7a and will also possess Rab-GAP activity. Furthermore, the Rab-GAP activity experiments were performed to determine whether the purified protein was the shark TBC1D15.

In pull-down experiments, MBP-Rab7a-WT/Q67L (GTP-bound state, active type) and T22N (GDP-bound state, inactive type) fusion proteins were expressed and purified to interact with shark TBC1D15. The results indicated that shark TBC1D15 could associate with Rab7a. The highest degree of binding was with Rab7a-Q67L, though the Rab7a-T22N type also showed a small amount of association. TBC1D15 easily associated with Rab7a-Q67L, which might promote Rab7a-GTP hydrolysis. Rab7a-T22N, a mimic of the GDP-bound form, may not be required to provide GAP activity. To further verify the changes of GTP hydrolysis due to GAP activity, we used a phosphate assay kit (EnzChek^®^ Phosphate Assay Kit, Invitrogen, E6646, Eugene, OR, USA) to detect the Pi content with GTP-GDP exchange. During the enzymatic reaction, the Pi content of GTP release was gradually increased and, therefore, the absorbance value of A_360_ increased. In comparison with the control groups, the Pi content was significantly increased in the experimental group, indicating that shark TBC1D15 exhibits good Rab-GAP activity for Rab7a and promotes GTP hydrolysis.

The current findings show that the TBC1D15 gene from *Chiloscylliu*m contains six classic motifs, possesses Rab-GAP activity and contains the APSL peptide at the *N*-terminus. Previous studies have demonstrated that APSL is involved in immunoregulation, promotes hepatocyte growth, and repairs damaged islet β cells, therefore showing potential therapeutic effects for liver damage and type-2 diabetes. However, the mechanism by which APSL functions has not yet been explored. In future studies, we intend to investigate the role of shark TBC1D15 in the regulation of diabetes by elucidating the key molecular mechanisms of this process. The present study may provide avenues for developing anti-diabetic drugs from marine sources in the future.

## 4. Experimental Methods

### 4.1. Construction of the Expression Vector

The gene sequences of the active APSL peptide from regenerating shark liver were cloned into a cDNA library. The prokaryotic expression vector pET-28a-APSL, which contained the target gene, was constructed successfully and stored in our laboratory.

The TBC1D15 gene containing an ORF of 2088 bp was screened from the cDNA library with high-throughput sequencing of the transcriptome from the regenerating shark liver. Based on the gene sequence, the shark TBC1D15 gene and the normal human Rab7a gene (Gene ID: 7879) were synthesized by Jin Weizhi Biotechnology (http://www.genewiz.com.cn, Suzhou, China). Using Primer Premier 5 software, shark TBC1D15 gene primers complementary to the flanking sequences of the ORF with the *Bam*H I and *Not* I recognition sites were designed as follows:

Upstream: 5′-CACGGATCCATGCTGGTGGGTCCGATCGG-3′
Downstream: 5′-CCGCGGCCGCTTACCACTTGAAGCTCTTCTTCTTG-3′

The TBC1D15 gene was amplified by PCR and ligated into the pETduet-His-sumo expression vector, whereas the Rab7a gene was ligated into the pDuet-Biotin-MBP expression vector.

Using the overlapping PCR method, the Rab7-Q67L/T22N gene was obtained based on mutations in the full-length gene locus. The primers used in the overlapping PCR experiment were as follows:

Pf1-Q67L: 5′-GTCACAATGCAGATATGGGACACAGCAGGACTGGAACGG-3′
Pr1-Q67L: 5′-GCAGGACTGGAACGGTTCCAGTCTCTCGGTGTGGCCTTC-3′
Pf2-T22N: 5′-GGAGATTCTGGAGTCGGGAAGAACTCACTC-3′
Pr2-T22N: 5′-GGGAAGAACTCACTCATGAACCAGTATGTG-3′

### 4.2. Purification of Recombinant Proteins

The recombinant plasmid pETduet-His-Sumo-TBC1D15 was transformed into *E. coli* BL21 (DE3) competent cells, which were incubated at 37 °C in liquid LB culture media containing 50 μg/mL ampicillin. The expression of the His-tag fusion protein was induced with the addition of IPTG (isopropylthio-β-d-galactoside) to a final concentration of 0.1 mM at an A_600_ of 0.6 before 20 h of incubation at 16 °C. *E. coli* cells were collected and resuspended in 35 mL of His-buffer A (25 mM imidazole, 200 mM NaCl, 20 mM Tris, 10% glycerol, pH 8.0) and then broken by high pressure. After centrifugation at 14,000 rpm for 20 min, the supernatant was incubated at 4 °C for 1 h with 2 mL of nickel-nitrilotriacetic acid (Ni-NTA) agarose and washed with five successive washes of 40 mL of His-buffer A. The fusion protein was eluted with 50% His-buffer A and 50% His-buffer B (500 mM imidazole, 200 mM NaCl, 20 mM Tris, 10% glycerol, pH 8.0). The protein concentrations were determined using the Bradford method. The purified protein was sub-packaged using standard procedures and stored at −20 °C.

The MBP fusion proteins were also expressed in *E. coli* and purified using a method similar to that described above for the His-tag fusion protein but with an amylose resin instead of Ni-NTA. The MBP fusion proteins were purified and eluted with 10 mM maltose solution in the MBP-buffer A (200 mM NaCl, 20 mM Tris, 10% glycerol, 10 mM maltose, pH 8.0).

### 4.3. Protein-Protein Interaction Assays in Vitro (Pull-Down Assays)

Protein interaction assays *in vitro* were performed after purification of the MBP and His fusion proteins. The samples were dialyzed to remove imidazole from the purified His fusion protein. For each group of experiments, 800 μL (150 μg) of His-TBC1D15 fusion proteins (bait protein) were added to pre-equilibrated Ni-NTA agarose and the beads were incubated at 4 °C. After approximately 1 h, the flow-through was collected, labeled “bait flow-through”, and placed on ice. The beads were washed with His-buffer A five times, and the flow-through was collected and labeled “bait wash”. Then, the MBP-Rab7-WT/T22N/Q67L (prey protein, 30 µg) was mixed with Ni-NTA agarose bound to His-sumo-TBC1D15 and incubated at 4 °C for 2 h. The flow-through was collected, labeled “prey flow-through”, and placed on ice. Next, the beads were washed five times with His-buffer A, and the flow-through was collected and labeled “prey wash”. Eventually, the proteins were eluted with high-imidazole buffer and prepared for separation by gel electrophoresis using two 12% SDS-PAGE gels. The proteins were separately transferred from the gels to PVDF. The prey MBP fusion proteins were detected using a polyclonal antibody against MBP (Proteintech, Wuhan, China), and the His fusion proteins were detected using a polyclonal antibody against His (Proteintech, Wuhan, China).

### 4.4. GTPase Activity Assay

GTPase activity assays were carried out according to the instructions of the EnzChek^®^ Phosphate Assay Kit (Invitrogen E6646, Eugene, OR, USA), which provides a fast and sensitive spectrophotometric method for the continuous detection of phosphate released in enzymatic reactions. In brief, 10 μL each of the purified Rab7 (700 nM) and TBC1D15 (300 nM) fusion proteins were mixed in a 1 mL reaction volume, which contained 50 μL of 20× reaction buffer, 200 μL of MESG substrate solution, 10 μL of purine nucleoside phosphorylase (1 U) and 720 μL of ddH_2_O. After incubation at 22 °C for 10 min, 1 μL of 2 mM GTP (Sigma G8877, St. Louis, MO, USA) was added to the above reaction volume and mixed well. Then, the A_360_ was recorded using a spectrophotometer every 5 min to determine the absorbance of the reaction as a function of time. Finally, 20 μL of buffer (instead of TBC1D15 + Rab7a) was used as a parallel control group.

## 5. Conclusions

In this study, the shark TBC1D15 gene was obtained from a cDNA library of regenerating shark liver. The total length of this sequence is 4213 bp and contains an ORF of 2088 bp. This protein contains the TBC domain with six motifs (A–F). In addition, it can activate Rab-GAP activity toward Rab7a. Our study is the first to report shark TBC1D15 as a new member of TBC domain family member 15, and it harbors APSL in its *N*-terminus. The biological role of this protein will be explored in the area of diabetes regulation, providing leads for developing successful anti-diabetic drugs from marine sources.

## References

[1] Ortis F., Naamane N., Flamez D., Ladriere L., Moore F., Cunha D.A., Colli M.L., Thykjaer T., Thorsen K., Orntoft T.F. (2010). Cytokines interleukin-1beta and tumor necrosis factor-alpha regulate different transcriptional and alternative splicing networks in primary beta-cells. Diabetes.

[2] Jiang Y., Zhao M.Y., An W. (2011). Increased hepatic apoptosis in high-fat diet-induced NASH in rats may be associated with downregulation of hepatic stimulator substance. J. Mol. Med..

[3] LaBrecque D.R., Steele G., Fogerty S., Wilson M., Barton J. (1987). Purification and physical-chemical characterization of hepatic stimulator substance. Hepatology.

[4] Huang F.J., Lv Z.B., Li Q., Wei L.J., Zhang L., Wu W.T. (2005). Study on hepatoprotective effect of peptide S-8300 from shark liver. World J. Gastroenterol..

[5] OU Y., Li Q. (2003). Purification and characterization of hepatocyte regeneration stimulatory factor from shark liver. J. Chin. Pharm. Sci..

[6] Lv Z., Ou Y., Li Q., Zhang W., Ye B., Wu W. (2009). Expression, purification and bioactivities analysis of recombinant active peptide from shark liver. Mar. Drugs.

[7] Liu Y., Chen Y., Chen J., Zhang W., Sheng Q., Chen J., Yu W., Nie Z., Zhang Y., Wu W. (2013). A shark liver gene-derived active peptide expressed in the silkworm, *Bombyx mori*: Preliminary studies for oral administration of the recombinant protein. Mar. Drugs.

[8] Huang F.J., Wu T. (2010). Purification and characterization of a new peptide (S-8300) from shark liver. J. Food Biochem..

[9] Huang F., Wu W. (2005). Antidiabetic effect of a new peptide from *Squalus mitsukurii* liver (S-8300) in streptozocin-induced diabetic mice. J. Pharm. Pharmacol..

[10] Huang F., Wu W. (2005). Antidiabetic effect of a new peptide from *Squalus mitsukurii* liver (S-8300) in alloxan-diabetes. Clin. Exp. Pharmacol. Physiol..

[11] Liu Y., Gao Z., Guo Q., Wang T., Lu C., Chen Y., Sheng Q., Chen J., Nie Z., Zhang Y. (2014). Anti-diabetic effects of CTB-APSL fusion protein in type 2 diabetic mice. Mar. Drugs.

[12] Neuwald A.F. (1997). A shared domain between a spindle assembly checkpoint protein and Ypt Rab-specific GTPase-activators. Trends Biochem. Sci..

[13] Fukuda M. (2011). TBC proteins: GAPs for mammalian small GTPase Rab?. Biosci. Rep..

[14] Zhang X.M., Walsh B., Mitchell C.A., Rowe T. (2005). TBC domain family, member 15 is a novel mammalian Rab GTPase-activating protein with substrate preference for Rab7. Biochem. Biophys. Res. Commun..

[15] Vind B.F., Pehmoller C., Treebak J.T., Birk J.B., Hey-Mogensen M., Beck-Nielsen H., Zierath J.R., Wojtaszewski J.F., Hojlund K. (2011). Impaired insulin-induced site-specific phosphorylation of TBC1 domain family, member 4 (TBC1D4) in skeletal muscle of type 2 diabetes patients is restored by endurance exercise-training. Diabetologia.

[16] Sano H., Kane S., Sano E., Miinea C.P., Asara J.M., Lane W.S., Garner C.W., Lienhard G.E. (2003). Insulin-stimulated phosphorylation of a Rab GTPase-activating protein regulates GLUT4 translocation. J. Biol. Chem..

[17] Cartee G.D. (2015). Roles of TBC1D1 and TBC1D4 in insulin- and exercise-stimulated glucose transport of skeletal muscle. Diabetologia.

[18] Gabernet-Castello C., O’Reilly A.J., Dacks J.B., Field M.C. (2013). Evolution of Tre-2/Bub2/Cdc16 (TBC) Rab GTPase-activating proteins. Mol. Biol. Cell.

[19] Mellouk N., Weiner A., Aulner N., Schmitt C., Elbaum M., Shorte S.L., Danckaert A., Enninga J. (2014). Shigella subverts the host recycling compartment to rupture its vacuole. Cell Host Microbe.

[20] Pan X., Eathiraj S., Munson M., Lambright D.G. (2006). TBC-domain GAPs for Rab GTPases accelerate GTP hydrolysis by a dual-finger mechanism. Nature.

[21] Albert S., Will E., Gallwitz D. (1999). Identification of the catalytic domains and their functionally critical arginine residues of two yeast GTPase-activating proteins specific for Ypt/Rab transport GTPases. EMBO J..

[22] Peralta E.R., Martin B.C., Edinger A.L. (2010). Differential effects of TBC1D15 and mammalian Vps39 on Rab7 activation state, lysosomal morphology, and growth factor dependence. J. Biol. Chem..

[23] Feldman D.E., Chen C., Punj V., Machida K. (2013). The TBC1D15 oncoprotein controls stem cell self-renewal through destabilization of the Numb-p53 complex. PLoS ONE.

[24] Onoue K., Jofuku A., Ban-Ishihara R., Ishihara T., Maeda M., Koshiba T., Itoh T., Fukuda M., Otera H., Oka T. (2012). Fis1 acts as a mitochondrial recruitment factor for TBC1D15 that is involved in regulation of mitochondrial morphology. J. Cell Sci..

[25] Zaid H., Antonescu C.N., Randhawa V.K., Klip A. (2008). Insulin action on glucose transporters through molecular switches, tracks and tethers. Biochem. J..

[26] Larance M., Ramm G., Stockli J., van Dam E.M., Winata S., Wasinger V., Simpson F., Graham M., Junutula J.R., Guilhaus M. (2005). Characterization of the role of the Rab GTPase-activating protein AS160 in insulin-regulated GLUT4 trafficking. J. Biol. Chem..

[27] Holman G.D., Sakamoto K. (2008). Regulating the motor for GLUT4 vesicle traffic. Cell Metab..

[28] Ishikura S., Koshkina A., Klip A. (2008). Small G proteins in insulin action: Rab and Rho families at the crossroads of signal transduction and GLUT4 vesicle traffic. Acta Physiol..

[29] Peck G.R., Chavez J.A., Roach W.G., Budnik B.A., Lane W.S., Karlsson H.K., Zierath J.R., Lienhard G.E. (2009). Insulin-stimulated phosphorylation of the Rab GTPase-activating protein TBC1D1 regulates GLUT4 translocation. J. Biol. Chem..

[30] O’Gorman D.J., Karlsson H.K., McQuaid S., Yousif O., Rahman Y., Gasparro D., Glund S., Chibalin A.V., Zierath J.R., Nolan J.J. (2006). Exercise training increases insulin-stimulated glucose disposal and GLUT4 (SLC2A4) protein content in patients with type 2 diabetes. Diabetologia.

[31] Sakamoto K., Holman G.D. (2008). Emerging role for AS160/TBC1D4 and TBC1D1 in the regulation of GLUT4 traffic. Am. J. Physiol. Endocrinol. Metab..

[32] Zhang J., Liu Y., Zhang X., Pan J., Nie Z., Zhang W., Yu W., Chen J., Liu L., Li J. (2013). The identification of microRNAs in the whitespotted bamboo shark (*Chiloscyllium plagiosum*) liver by Illumina sequencing. Gene.

[33] Lu C., Zhang J., Nie Z., Chen J., Zhang W., Ren X., Yu W., Liu L., Jiang C., Zhang Y. (2013). Study of microRNAs related to the liver regeneration of the whitespotted bamboo shark, *Chiloscyllium plagiosum*. BioMed. Res. Int..

[34] Mayer A.M., Rodriguez A.D., Taglialatela-Scafati O., Fusetani N. (2013). Marine pharmacology in 2009–2011: Marine compounds with antibacterial, antidiabetic, antifungal, anti-inflammatory, antiprotozoal, antituberculosis, and antiviral activities; affecting the immune and nervous systems, and other miscellaneous mechanisms of action. Mar. Drugs.

[35] Ma Y., Mi Y.J., Dai Y.K., Fu H.L., Cui D.X., Jin W.L. (2013). The inverse F-BAR domain protein srGAP2 acts through srGAP3 to modulate neuronal differentiation and neurite outgrowth of mouse neuroblastoma cells. PLoS ONE.

